# TP901-1 Phage Recombinase Facilitates Genome Engineering in *Drosophila melanogaster*

**DOI:** 10.1534/g3.119.0002

**Published:** 2019-01-29

**Authors:** Roumen Voutev, Richard S. Mann

**Affiliations:** Department of Biochemistry and Molecular Biophysics and Systems Biology, Columbia University, Zuckerman Mind Brain Behavior Institute, 3227 Broadway, Quad 9A, New York, NY 10027

**Keywords:** TP901-1, site-directed recombinase, cassette exchange, CRISPR/Cas9, genome engineering, *Drosophila melanogaster*

## Abstract

Molecular biology techniques have a large impact on biomedical research and the availability of diverse tools to perform genome manipulations advances the ease of executing complicated genetic research. Here, we introduce in the fruit fly another such tool by harnessing the phage recombinase TP901-1 to perform site-directed recombination that leads to recombinase-mediated cassette exchange (RMCE). The TP901-1 system complements already existing recombination systems and enhances genome engineering in the fruit fly and other organisms.

Recombinase-mediated cassette exchange (RMCE) is a powerful molecular biology technique that can enhance genome manipulations, including CRISPR/Cas9 genome editing. Efficient RMCE in *Drosophila melanogaster* has been performed using diverse recombination systems and is an established molecular genetics tool (Horn and Handler 2005; Oberstein *et al.* 2005; Bateman *et al.* 2006; Voutev and Mann 2017). The irreversibility of the site-directed recombination is critical in RMCE because it ensures that once recombination occurs the edited genomic locus is ‘locked’ in a desired state. Heterospecific *FRT* or *lox* sites are normally employed to achieve directionality and conditional irreversibility when the *Flp/FRT* or the *Cre/lox* systems are used for RMCE in the fruit fly (Horn and Handler 2005; Oberstein *et al.* 2005). While in the case of serine integrase systems, such as those based on the ΦC31 and Bxb1 recombinases, the conversion of their cognate recognition attB/attP sites to attL/attR sites during recombination (Thorpe and Smith 1998; Ghosh *et al.* 2003) leads to irrevocable cassette exchange of, for example, attP-flanked loci in *Drosophila* (Bateman *et al.* 2006; Voutev and Mann 2017). In order to broaden the spectrum of available irreversible site-directed recombinase systems in the fruit fly, we show that the TP901-1 lactococcal phage recombinase (Christiansen *et al.* 1996) could perform successful recombination and RMCE by using minimal TP901-1 attB/attP sites.

## Methods and Materials

The original TP901-1 recombinase cDNA was PCR amplified from plasmid pG35-TP901.2 (a gift from Dr. James Thomson; (Thomson and Ow 2006)) and inserted in vector pRVV210 (Addgene ID#87628; (Voutev and Mann 2017)) in place of the Bxb1 recombinase cDNA, resulting in construct *vasa-TP901-1-nos*. The fruit fly codon-optimized version of TP901-1 recombinase gene was synthesized based on the analysis of the IDT Codon Optimization Tool (http://www.idtdna.com/CodonOpt; Integrated DNA Technologies Inc., Skokie, IL). The resulting cDNA, TP901-1^FlyOpt^, was inserted in place of the original cDNA to generate *vasa-TP901-1^FlyOpt^-nos* and additional constructs were created through PCR amplification by adding the SV40 large T-antigen NLS sequence (Kalderon *et al.* 1984) either to the N-terminus or to the C-terminus of TP901-1^FlyOpt^. A *yellow* (*y+*) selectable marker was added to these vectors afterward. Since the most efficient TP901-1^FlyOpt^ recombinase version was the one with an N-terminally tagged NLS, the vector carrying it (pRVV655; Addgene ID#119019) was deposited in Addgene, Cambridge, MA. Different fly strains carrying *vasa-NLS-TP901-1^FlyOpt^-nos* were generated by inserting the construct in landing sites ZH-2A, ZH-22A, and ZH-86Fa on chromosomes X, II, and III, respectively (Bischof *et al.* 2007). These fly strains were deposited in Bloomington Drosophila Stock Center, Bloomington, IN.

**Figure 1:**
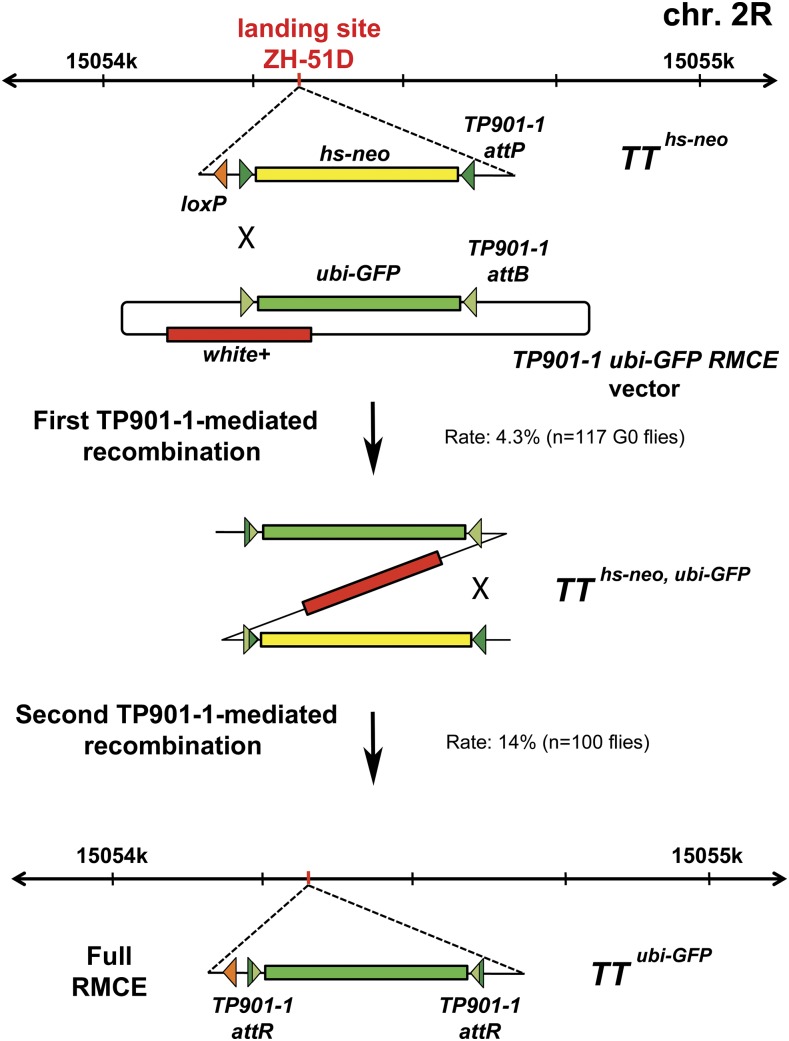
Schematic representation of the ZH-51D landing site locus on chromosome 2R and its two-step genome engineering from *TT^hs-neo^* allele to *TT^ubi-GFP^* allele by using TP901-1-catalyzed recombinase-mediated cassette exchange (RMCE). Dark green represents a 65bp TP901-1 attP site; olive green represents a 61bp TP901-1 attB site; bright green represents a *ubi-GFP* cassette.

The 65 bp minimal TP901-1 recombinase attP site (TCCAACTCGCTTAATTGCGAGTTTTTATTTCGTTTATTTCAATTAAGGTAACTAAAAAACTCCTT; (Thomson and Ow 2006)) was introduced in vector pRVV598 (Addgene ID#87629, (Voutev and Mann 2017)) in forward and reverse orientation (Figure 1), flanking a *hs-neo* cassette (Steller and Pirrotta 1985) and replacing the Bxb1 recombinase attP sites in vector pRVV598 (Voutev and Mann 2017). The *hs-neo* cassette consists of a *heat-shock-protein-70* promoter driving neomycin resistant gene (Steller and Pirrotta 1985) but drug selection was not performed in this study. A loxP site was introduced 5′ of this cassette and the resulting vector was used for injection and integration in landing site ZH-51D to subsequently generate a clean allele, *TT^hs-neo^*.

The 61 bp minimal TP901-1 recombinase attB site (CTGATAATTGCCAACACAATTAACATCTCAATCAAGGTAAATGCTTTTTGCTTTTTTTGCC; (Thomson and Ow 2006)) was introduced in vector pRVV601 (Addgene ID#87632; (Voutev and Mann 2017)) in forward and reverse orientation, flanking an MCS and replacing the Bxb1 recombinase attB sites in vector pRVV601, resulting in an TP901-1 attB RMCE vector (pRVV610; Addgene ID#119018). Shorter TP901-1 attB/attP pairs have successfully been used for recombination in bacteria (Breüner *et al.* 2001; Stoll *et al.* 2002) but since the recombination efficiency of TP901-1 in the fruit fly was not very high (see below), we refrained from testing shorter TP901-1 attB/attP sites than the ones described by thomson and ow 2006.

The *TP901-1 ubi-GFP RMCE* vector (Figure 1) was created by PCR amplifying a *ubi-GFP* cassette and placing it in the MCS of pRVV610. Plasmid DNA, maps, and complete vector sequences of the above and other useful TP901-1 system vectors are made available at Addgene (www.addgene.org); Addgene IDs: 119014-119019.

### Data availability

The authors state that all data necessary for confirming the conclusions presented in the article are represented fully within the article.

## Results and Discussion

Similar to our previous RMCE technique studies (Voutev and Mann 2017; Voutev and Mann 2018), we introduced in landing site ZH-51D a *hs-neo* cassette flanked by minimal inverted TP901-1 attP sites (Figure 1; Methods and Materials), using standard ΦC31-mediated integration (Bischof *et al.* 2007). A loxP site that we positioned upstream of the *hs-neo* cassette allowed us to excise all intervening vector sequences (Bischof *et al.* 2007) and to create a clean TP901-1 attP-flanked *hs-neo* allele that we called *TT^hs-neo^* (Figure 1).

Next, we generated a fly strain that expressed the TP901-1 phage recombinase in the fly germ line by placing its cDNA under the control of the *vasa* promoter and *nanos* 3′UTR (*vasa-TP901-1-nos*) and introduced this construct into landing site ZH-86Fa (Bischof *et al.* 2007) through ΦC31-mediated transgenesis.

We also created an RMCE vector that contains *ubiquitin-GFP* (*ubi-GFP*) cassette flanked by minimal inverted TP901-1 recombinase attB sites (Figure 1; Methods and Materials). The *TP901-1 ubi-GFP RMCE* vector is marked by *white* (*w+*) (Figure 1) that allows the differentiation between RMCE and integration events in our experimental design.

Previously (Voutev and Mann 2018), we found that the ΦC31 integrase performs most efficient recombination when it is expressed in the fly germ line in an established strain that contains two copies of the transgene driving its expression (most likely because of increased dosage of the recombinase). Analogously, we decided to test if the TP901-1 recombinase could perform RMCE when the transgene *vasa-TP901-1-nos [ZH-86Fa]* is homozygous. We established a strain, *TT^hs-neo^/CyO*; *vasa-TP901-1-nos [ZH-86Fa]*, and injected embryos laid by it with the *TP901-1 ubi-GFP RMCE* vector at 250 ng/µl concentration. We raised the resulting larvae at 25° and crossed individual fertile adults to *yw* flies. Surprisingly, these injections did not lead to any RMCE or integration events of the *TP901-1 ubi-GFP RMCE* vector even though we injected more than 1000 embryos that produced ∼350 fertile adults.

Since the TP901-1 recombinase has been shown to function *in vitro* and in other eukaryotic systems (Stoll *et al.* 2002; Thomson and Ow 2006), it was unlikely that it required additional factors to perform recombination in the fruit fly. However, the optimal temperature for TP901-1 function is 30-35° (Stoll *et al.* 2002), which is above the standard temperatures for fly husbandry and is closer to temperatures normally used in fly heat-shocks. To test if higher temperatures would improve TP901-1 recombination performance, we injected 800 *TT^hs-neo^/CyO*; *vasa-TP901-1-nos [ZH-86Fa]* embryos, with *TP901-1 ubi-GFP RMCE* vector and raised the resulting larvae and crossed the adults to *yw* flies at 29.5°. This experiment also did not lead to any positive RMCE or integration events.

The above results suggested that there might be some other reason why the TP901-1 recombinase does not function in flies. Since we have previously used the same vector backbone and drivers to express the Bxb1 recombinase in *Drosophila* (Voutev and Mann 2017), we reasoned that the problem did not lie in the expression levels driven by the *vasa* promoter and the *nos* 3′UTR. Therefore, we looked more closely at the original cDNA of TP901-1 and found that it uses unfavorable codons for the fruit fly (*e.g.*, 22 ACU (Thr), 36 AAA (Lys), 12 UUA (Leu), 12 UCA (Ser), 11 AGA (Arg)). Therefore, we codon-optimized the TP901-1 recombinase cDNA to favor translation in the fruit fly (Methods and Materials) and created a new construct (*vasa-TP901-1^FlyOpt^-nos*) that we introduced in landing site ZH-86Fa. In addition, we tested if adding the SV40 large T-antigen nuclear localization signal (Kalderon *et al.* 1984) to either the N- or the C-terminus would improve the function of the TP901-1 recombinase in flies. We created two additional vectors and transgenes: *vasa-NLS-TP901-1^FlyOpt^-nos [ZH-86Fa]* and *vasa-TP901-1^FlyOpt^-NLS-nos [ZH-86Fa]*. The strains carrying these transgenes did not exhibit any noticeable toxicity to the flies.

We tested each of the optimized TP901-1 recombinase transgenes by injecting with the *TP901-1 ubi-GFP RMCE* vector ∼400 embryos produced by each of the following strains: *TT^hs-neo^/CyO*; *vasa-TP901-1^FlyOpt^-nos [ZH-86Fa]*, *TT^hs-neo^/CyO*; *vasa-NLS-TP901-1^FlyOpt^-nos [ZH-86Fa]* and *TT^hs-neo^/CyO*; *vasa-TP901-1^FlyOpt^-NLS-nos [ZH-86Fa]*. We performed these experiments at standard temperature, 25°. The optimization of the TP901-1 recombinase improved its performance because each of the injection schemes produced integration events through recombination between one of the TP901-1 attB/attP pairs (GFP-positive, *w+* flies). However, no full RMCE events (GFP-positive, *w*- flies) were observed. *NLS-TP901-1^FlyOpt^* was the most efficient recombinase and produced 4.3% (n = 117) G0 flies that segregated progeny with integration events. *TP901-1^FlyOpt^* and *TP901-1^FlyOpt^-NLS* were less efficient and produced 1.2% (n = 83) and 1% (n = 97) G0s, respectively, which segregated progeny with integration events.

Previously, we showed that during cassette exchange, integration events catalyzed by either the ΦC31 or the Bxb1 integrases could be converted to complete RMCE events through intramolecular recombination between intact attB/attP pairs left in the genome (Voutev and Mann 2017; Voutev and Mann 2018). Similarly, we wanted to test if the optimized TP901-1 recombinase could perform intramolecular excisions. We maintained the *TT^hs-neo,ubi-GFP^* alleles (Figure 1), resulting from the integration events, in the background of optimized TP901-1 recombinase in its three versions: *TT^hs-neo^*^,^ *^ubi-GFP^/CyO*; *vasa-TP901-1^FlyOpt^-nos/MKRS*, *TT^hs-neo^*^,^ *^ubi-GFP^/CyO*; *vasa-NLS-TP901-1^FlyOpt^-nos/MKRS* and *TT^hs-neo^*^,^ *^ubi-GFP^/CyO*; *vasa-TP901-1^FlyOpt^-NLS-nos/MKRS*. We crossed males with these genotypes to *yw* females and asked if, and at what rate, intramolecular excision could be observed in the resulting non-*CyO* progeny by absence of the *w+* marker in the eyes. All three optimized versions of the TP901-1 recombinase catalyzed intramolecular recombination (Figure 1) and *NLS-TP901-1^FlyOpt^* outperformed the other two versions since the males carrying it produced 14/100 *w*- GFP-positive flies with complete RMCE. The other two optimized versions of TP901-1 catalyzed intramolecular recombination at equal rates since males in each case produced 7/100 flies that were *w*- GFP-positive. We sequence-verified four of these recombination events to confirm that the RMCE occurred correctly. As expected, we found that the *ubi-GFP* cassette was inserted in either forward or reverse orientation.

**Figure 2:**
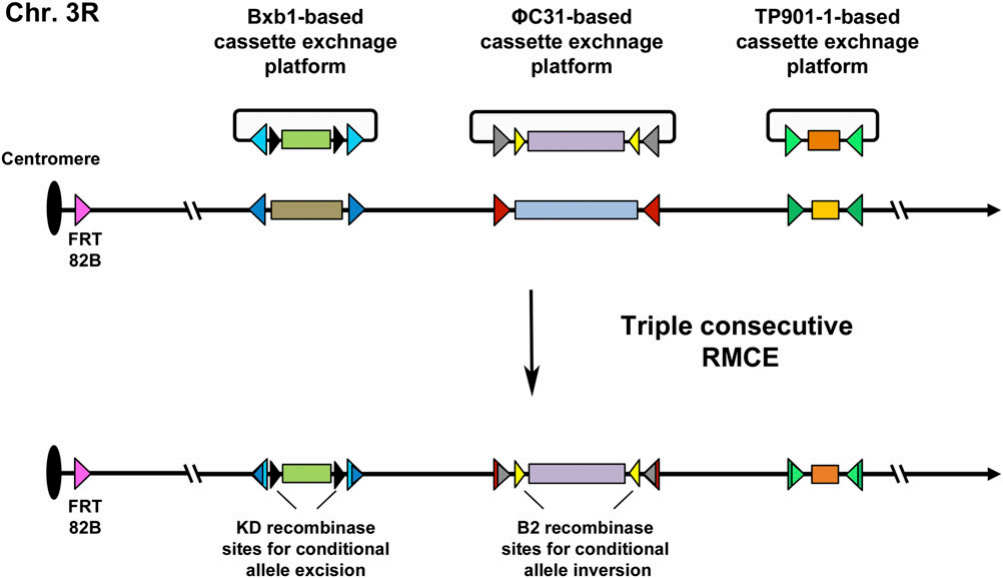
Schematized example of three engineered platforms for cassette exchange on chromosome 3R, based on the Bxb1, ΦC31 and TP901-1 recombinase systems. Platforms are generated on the genetic background of FRT82B in order to perform clonal analysis by using the Flp/FRT recombination system. Recognition sites for the KD recombinase (represented with black triangles) and the B2 recombinase (represented with yellow triangles) or other site-directed recombinases could be introduced to flank particular sequences in order to conditionally excise or invert specific alleles. The Bxb1 attP/attB sites are represented with dark/light blue triangles, respectively; the ΦC31 attP/attB sites are represented with maroon/gray triangles, respectively; the TP901-1 attP/attB sites are represented with dark/light green, respectively.

The successful replacement of the *hs-neo* cassette with *ubi-GFP* through two-step TP901-1^FlyOpt^-catalyzed recombination (Figure 1) marks the introduction of yet another site-directed recombinase system in the repertoire of *Drosophila melanogaster* molecular genetics tools. Despite the fact that TP901-1^FlyOpt^ was not observed to catalyze complete RMCE events in one step and required a second intramolecular excision step, it is nevertheless useful in genome engineering of loci where multiple irreversible recombinase systems are required. For example, three genome elements of interest could be engineered on the same chromosome arm using three different recombinase systems (ΦC31, Bxb1, or TP901-1) and studied simultaneously (Figure 2). These genome elements could be different ‘shadow’ enhancers, paralogous genes, exons, promoters, 3′UTRs, insulators, snRNAs, etc., that are positioned too far from each other to be studied on a single platform for cassette exchange and too close to each other to be easily recombined with each other. The resulting triple platform for cassette exchange would allow thorough combinatorial investigation of different versions of these genome elements. In addition, since the Flp/FRT system is normally used for clonal analysis in the fruit fly (Xu and Rubin 1993; Lee and Luo 1999), which renders it unfeasible to use for creation of platforms for cassette exchange if subsequent clonal analysis of the generated alleles is required, the engineered triple platform for cassette exchange could easily be combined with standard FRT sites (Xu and Rubin 1993) that are positioned close to the centromere (Figure 2). Also, sites for other available reversible recombination systems, such as those of the KD, B2, B3 or R recombinases (Nern *et al.* 2011) could be introduced in desired manner within the replacement cassettes in order to create conditional alleles where a specific studied genome element could be inverted or removed upon expression of the corresponding recombinase depending on the directionality of the introduced cognate sites (Figure 2).

Usage of irreversible recombinase systems (ΦC31, Bxb1, TP901-1) for creation of platforms for cassette exchange prevents aberrant reversion of alleles during the RMCE steps and the respective recombinases could be expressed again in the background of such alleles for different purposes. Reversible recombinase systems, on the other hand, are very suitable for excision/inversion of cassettes in post-engineering allele-manipulation steps and generation of stochastic clones. Alleles containing reversible recombinase sites are not suitable for subsequent expression of the corresponding recombinase for other genetic purposes as chromosomal rearrangements may occur. For example, engineered alleles generated through RMCE by using the ΦC31, Bxb1, or TP901-1 systems could be readily combined with the Flybow (Hadjieconomou *et al.* 2011) and dBrainbow (Hampel *et al.* 2011) cell labeling techniques, which are based on the Flp/FRT and Cre/lox recombination systems, respectively, without concerns that aberrant recombination might occur.

The introduction of the TP901-1 recombinase system as a genome engineering tool in the fruit fly could facilitate combinatorial genomic research where creation of multiple platforms for cassette exchange (Figure 2) could allow generation of allelic series that greatly augments the research value of a particular genetic study. Similar optimizations of the TP901-1 recombinase could also be done for other organisms in order to render the system useful in diverse species.
